# Risk of type 2 diabetes mellitus associated with lipid-related metabolic indicators: a diabetes family cohort study in China

**DOI:** 10.1186/s12902-026-02484-8

**Published:** 2026-08-12

**Authors:** Haowei Li, Yilu Huang, Donghui Yang, Zizhu Li, Liangyou Wang, Chaowei Fu

**Affiliations:** 1 School of Public Health, NHC Key Laboratory of Health Technology Assessment, Key Laboratory of Public Health Safety, Fudan University, Shanghai, 200032 China; 2 Department of Non-Communicable Chronic Disease Control and Prevention, Taizhou Center for Disease Control and Prevention (Taizhou Health Supervision Institute), Taizhou, 318000 China

**Keywords:** Metabolic indicators, Type 2 diabetes mellitus, Family-based cohort study, Triglycerides

## Abstract

**Objective:**

To evaluate the associations between lipid-related metabolic indicators and incident type 2 diabetes mellitus (T2DM).

**Methods:**

A diabetes family cohort study of 3,726 participants was conducted in Taizhou from May to October 2021 to track incident T2DM cases. Lipid metabolism indicators included triglycerides (TG), total cholesterol (TC), high-density lipoprotein cholesterol (HDL-C), low-density lipoprotein cholesterol (LDL-C), triglyceride-glucose (TyG) and its related indices, Chinese Visceral Adiposity Index (CVAI), and homeostasis model assessment of insulin resistance (HOMA-IR). Mixed-effects Cox proportional hazards models, within-family Cox models, and quartile analyses were performed to examine the associations between these indicators and incident T2DM.

**Results:**

Among 1,257 participants without diabetes at baseline, 68 developed incident T2DM during the average follow-up of 3.92 years. Higher levels of TyG (HR = 1.758, 95% CI: 1.182–2.616), TyG-BMI (HR = 1.007, 95% CI: 1.002–1.013), TyG-WHtR (HR = 2.108, 95% CI: 1.468–3.027), TyG-WC (HR = 1.005, 95% CI: 1.003–1.007), CVAI (HR = 1.013, 95% CI: 1.006–1.020), and HOMA-IR (HR = 1.195, 95% CI: 1.038–1.376) were significantly associated with an increased risk of incident T2DM, whereas traditional lipid indicators showed no significant associations. These findings were consistent in within-family analyses. Furthermore, dose-response relationships were observed between TyG, TyG-WHtR, TyG-WC, CVAI and incident T2DM.

**Conclusions:**

In conclusion, TyG-related indices, CVAI, and HOMA-IR were significantly associated with incident T2DM in this diabetes family-based cohort. These composite indicators may be useful for the early identification of high-risk individuals and for informing targeted prevention strategies.

**Clinical trial number:**

Not applicable.

**Supplementary Information:**

The online version contains supplementary material available at https://doi.org/10.1186/s12902-026-02484-8.

## Introduction

Type 2 diabetes mellitus (T2DM) is a major global public health challenge. According to the International Diabetes Federation, 589 million adults aged 20–79 years were living with diabetes worldwide in 2024, and this number was projected to rise to more than 850 million by 2050 [1]. T2DM imposes a substantial burden in terms of premature mortality and health-care expenditure worldwide [2]. China has the largest number of people living with diabetes globally, and the prevalence and disease burden of diabetes will rise up in the coming decades [3, 4]. Therefore, early identification of individuals at high risk of T2DM remains a key priority for the prevention and control.

Dyslipidemia and insulin resistance are central to the incident and development of T2DM. Evidence suggests that abnormal lipid metabolism is not merely a consequence of impaired glucose homeostasis, but may also contribute to insulin resistance and T2DM through lipotoxicity, chronic inflammation, oxidative stress, ectopic fat deposition, and β-cell dysfunction [5]. In this context, both traditional lipid parameters and lipid-related composite indicators may help capture metabolic abnormalities before the onset of T2DM. Recent cohort studies have emphasized that composite metabolic indicators integrating triglycerides, glucose, and adiposity may provide better discrimination for future T2DM risk than conventional single or non-integrated metabolic measures [6, 7]. Among these indicators, the triglyceride-glucose (TyG) index has been consistently associated with incident T2DM in different populations [8–10]. Cohort studies further suggested that TyG-related indices incorporating anthropometric measures, such as Triglyceride-glucose body mass index (TyG-BMI), Triglyceride-glucose waist-to-height ratio (TyG-WHtR) and Triglyceride-glucose waist circumference (TyG-WC), may improve risk stratification by jointly reflecting insulin resistance and obesity-related metabolic burden [11, 12]. Beyond TyG-related indices, visceral adiposity-related indicators have also gained increasing attention. Chinese cohort studies have shown that higher Chinese Visceral Adiposity Index (CVAI) levels, as well as longitudinal increases in CVAI, were associated with a higher incidence of cardiometabolic diseases, especially diabetes [13, 14]. Meanwhile, homeostasis model assessment of insulin resistance (HOMA-IR) is a widely used indicator of insulin resistance and has also been associated with incident T2DM risk [7, 15, 16]. Taken together, these findings suggested that composite metabolic indicators captured multiple dimensions of metabolic dysfunction and were consistently associated with future T2DM risk.

Family-based population studies may provide additional insights into the relationship between metabolic indicators and incident T2DM, as individuals with a family history of diabetes may share genetic susceptibility and familial environmental factors that influence both metabolic traits and diabetes risk [17, 18]. These shared familial factors may introduce residual confounding in conventional population-based cohorts, whereas family-based designs accounting for familial clustering may help determine whether metabolic indicators remain associated with T2DM risk beyond shared family-level background.

However, many previous studies have focused on a single marker or a limited set of indicators, making it difficult to directly compare traditional lipid measures with newer composite indices within the same population [19–21]. Also, although T2DM is influenced by both metabolic and familial factors, evidence is still limited on whether the associations of these indicators with incident T2DM persist after accounting for shared family background. Finally, data from family-based Chinese cohorts remained scarce, especially studies simultaneously evaluating conventional lipid markers, TyG-related indices, CVAI, and HOMA-IR. Therefore, this diabetes family cohort study aimed to investigate the associations between multiple lipid-related metabolic indicators and incident T2DM.

## Methods

### Study design and population

**Fig. 1:**
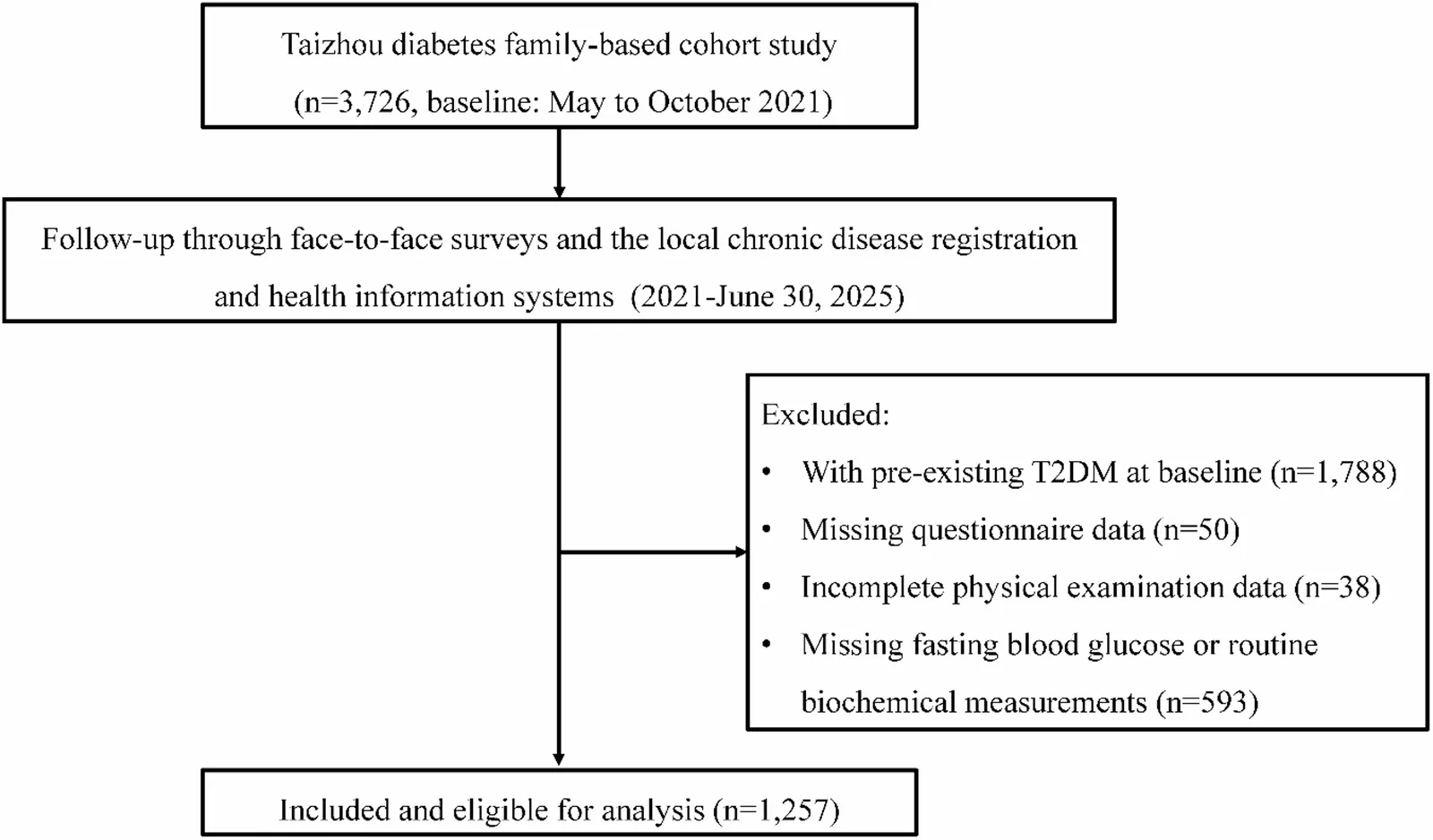
Flowchart of particpant recruitment

This diabetes cohort study was a family-based, epidemiological study of T2DM in Taizhou, Zhejiang Province, China. A stratified, randomized cluster sampling method was used to recruit participants from 6 districts of Taizhou. Specifically, confirmed eligible T2DM patients were first identified from the Taizhou Diabetes Management System as probands. Subsequently, their available family members, including first-, second-, and third-degree relatives, as well as spouses of probands and participating relatives, were invited to participate in the study. The baseline survey, conducted between May and October 2021, included 3,726 residents. The inclusion criteria (all of the following were required) were as follows: (1) the proband met the diagnostic criteria for T2DM, and at least one of their first-degree relatives (parents, siblings, or offspring) had T2DM, or the proband was diagnosed before the age of 35 years; (2) participants were registered local residents, had lived in the study area for at least one year, and had no plans to relocate within the next three years; (3) participants were conscious and able to cooperate with the questionnaire survey and physical examination; (4) participants voluntarily agreed to participate in the study and provided written informed consent. The exclusion criteria (any of the following) were: (1) non-local registered residents; (2) individuals planning to move out of the study area in the near future or those with long-term absence from the area; (3) individuals unable to provide informed consent or complete the questionnaire survey and physical examination; (4) individuals with a prior diagnosis of severe cardiovascular or cerebrovascular diseases, chronic kidney disease, other endocrine disorders, or malignancies. All participants provided written informed consent, and the study protocol was approved by the Institutional Review Board. After excluding participants with pre-existing T2DM at baseline (*n* = 1,788), missing questionnaire data (*n* = 50), incomplete physical examination data (*n* = 38), and missing fasting blood glucose or routine biochemical measurements (*n* = 593), a total of 1,257 eligible participants were included in the final analysis (Fig. 1).

### Questionnaire and anthropometric measurements

Data were collected using the family and individual questionnaires by trained health professionals at the local community health service centers. The family component collected basic information on family members, including demographic characteristics, chronic disease status, and kinship relationships. The individual component covered data on gender, age, education, occupation, annual household income per capita, personal and family medical history, as well as lifestyle factors such as smoking, alcohol consumption, tea consumption, physical activity, and dietary pattern. Anthropometric measurements were performed in local community health service centers using standardized protocols, including height, weight, waist circumference (WC), and hip circumference (HC), with each parameter measured twice and the mean value used in the analysis. The body mass index (BMI) was calculated as weight (kg) divided by height squared (m^2^).

### Laboratory measurements

Fasting venous blood samples were obtained after at least 8 h of overnight fasting. Serum concentrations of total cholesterol (TC) and triglycerides (TG) were measured using enzymatic methods, while high-density lipoprotein cholesterol (HDL-C) and low-density lipoprotein cholesterol (LDL-C) were determined using selective clearance methods (peroxidase-based method for HDL-C and surfactant-based method for LDL-C). Fasting plasma glucose (FPG) was measured after at least 8 h of fasting. Fasting insulin levels (FINS) were assessed using a chemiluminescence immunoassay. Hemoglobin A1c (HbA1c) and FPG were measured using standardized laboratory procedures but HbA1c was available for 16.87% of participants during follow-up. All laboratory analyses were conducted under strict quality control procedures.

### Definition of diabetes mellitus and follow-up

The study outcome was incident T2DM. T2DM was identified based on any of the following international diagnostic criteria: (1) self-reported T2DM; (2) fasting plasma glucose (FPG) ≥ 7.0 mmol/L; (3)glycated hemoglobin (HbA1c) ≥ 6.5%; (4) current use of glucose-lowering medications [22]. Outcome information was obtained through a combination of on-site follow-up and passive follow-up. The on-site follow-up was conducted from May to June 2025 and primarily involved the collection of biological samples and measurement of FPG and HbA1c. Passive follow-up was performed by linking participants’ personal identification numbers with the Taizhou Diabetes Surveillance Database and electronic health records of residents in each county or district. Through this linkage, information on FPG measurements from routine health examinations and incident diabetes diagnoses was obtained. Passive follow-up was completed on June 30, 2025.

### Definition of variables

Education was categorized as ≤ 9 and > 9 education years, based on China’s nine-year compulsory education policy. Marital status was classified as married or not. Smoking, alcohol, and tea consumption were classified as never and ever or current. Ever or current smoking was defined as smoking more than one cigarette per day for at least six months, whereas alcohol and tea consumption were defined as intake more than three times per week for at least six months. Physical activity was assessed using the International Physical Activity Questionnaire (IPAQ) and classified as low, moderate, or high levels [23]. Dietary patterns were evaluated based on dietary structure (predominantly meat-based, predominantly plant-based, or balanced), regularity of meals, and the intake of vegetables, fruits, oil, and salt. A composite score ranging from 0 to 8 was calculated to reflect overall dietary quality, with higher scores indicating healthier patterns. Participants were then classified into three groups: unbalanced (0–2), moderate (3–5), and balanced (6–8) [24].

Hypertension was defined as systolic blood pressure (SBP) ≥ 140 mmHg, or diastolic blood pressure (DBP) ≥ 90 mmHg, or self-reported hypertension, or a prior diagnosis before baseline, or current use of antihypertensive medications [25]. A family history of diabetes was defined as having at least one first-degree relative with diabetes. Abnormal lipid levels were defined as TC ≥ 5.2 mmol/L, TG ≥ 1.7 mmol/L, HDL-C < 1.0 mmol/L, and LDL-C ≥ 3.4 mmol/L. Dyslipidemia was defined as the presence of any of the above abnormalities, self-reported hyperlipidemia, a prior diagnosis before baseline, or current use of lipid-lowering medications [26]. TyG was calculated as ln [fasting triglycerides (mg/dL) × FPG (mg/dL)/2], as a surrogate marker of insulin resistance [27, 28]. TyG-related composite indices were further constructed to incorporate anthropometric measures. Specifically, TyG-BMI was calculated as TyG × BMI, TyG-WC as TyG × WC, and TyG-WHtR as TyG × WHtR [29, 30]. CVAI was calculated using the following sex-specific formulas [31]: Males: CVAI = − 267.93 + 0.68 × Age + 0.03 × BMI + 4.00 × WC + 22.00 × LgTG − 16.32 × HDL-C. Females: CVAI = − 187.32 + 1.71 × Age + 4.23 × BMI + 1.12 × WC + 39.76 × LgTG − 11.66 × HDL-C. Abnormal CVAI was defined as values greater than 106.900 in males and 115.018 in females [32]. HOMA-IR was calculated using the following formula [33]: FPG (mmol/L) × FINS (µU/mL) / 22.5.

### Statistical analysis

All data analyses were performed using R 4.4.1. Descriptive statistics were used to summarize the sociodemographic characteristics of the study population. Differences in baseline characteristics between participants with and without incident T2DM, and between those included in the final analysis and those excluded were examined using independent t-tests or Wilcoxon rank-sum tests for continuous variables and chi-square tests for categorical variables. To evaluate the association between lipid metabolism indicators and the risk of incident T2DM, mixed-effects Cox proportional hazards models were applied using the R package coxme, with family ID included as a random effect to account for intra-family correlation. The lipid metabolism indicators included TG, TC, HDL-C, LDL-C, TyG and its related parameters (TyG-BMI, TyG-WHtR, and TyG-WC), CVAI, and HOMA-IR. Also, within-family analyses were additionally performed using Cox proportional hazards models stratified by family ID to examine the robustness of the associations between lipid metabolism indicators and incident T2DM. The proportional hazards assumption of the Cox models was assessed using Schoenfeld residual tests, and no violations of the assumption were observed (all P values > 0.05). In addition, selected lipid metabolism indicators, including TyG, TyG-WHtR, TyG-WC, CVAI, and HOMA-IR, were categorized into quartiles, and mixed-effects Cox proportional hazards models were used to estimate HRs across quartiles, with the lowest quartile serving as the reference group. Tests for trend were conducted by including the median value of each quartile as a continuous variable in the models. A two-sided P value < 0.05 was considered statistically significant.

## Results

### Characteristics of participants

Among total of 1,257 participants without T2DM at baseline, 68 incident T2DM were identified during follow-up. Their mean age was 52.19 ± 16.02 years, with 768 (61.10%) females. Significant differences were observed between non-T2DM and T2DM groups in terms of age, marital status, alcohol consumption, abnormal TC and CVAI. The T2DM group was older, had higher proportions of individuals aged ≥ 60 years, and had higher proportions of married individuals, alcohol consumers, and those with abnormal total cholesterol and CVAI (Table 1). Compared with participants excluded because of incomplete physical examination or biochemical data, those included in the final analysis differed significantly in age, marital status, occupation status, hypertension, and alcohol consumption (Detailed in Supplementary Table 1).

**Table 1:** Baseline characteristics of the study population

Characteristics	Total (*n* = 1257)	Incident T2DM			
		No(*n* = 1189)	Yes(*n* = 68)	χ^2^/t	*P* value
Age, years	52.19 ± 16.02	64.27 ± 6.05	64.51 ± 5.35		< 0.001
< 60	881 (70.09)	849 (71.40)	32 (47.06)	17.042	< 0.001
≥ 60	376 (29.91)	340 (28.60)	36 (52.94)		
Gender, %				0.607	0.436
Male	489 (38.90)	459 (38.60)	30 (44.12)		
Female	768 (61.10)	730 (61.40)	38 (55.88)		
Education, years				0.025	0.875
< 9	852 (67.78)	807 (67.87)	45 (66.18)		
≥ 9	405 (32.22)	382 (32.13)	23 (33.82)		
Marital status, %				5.632	0.018
Married	1066 (84.81)	1001 (84.19)	65 (95.59)		
Not married	191 (15.19)	188 (15.81)	3 (4.41)		
Occupation, %				2.671	0.263
Mainly intellectual work	297 (23.63)	285 (23.97)	12 (17.65)		
Mainly physical labor	613 (48.77)	581 (48.86)	32 (47.06)		
Unemployment or others	347 (27.60)	323 (27.17)	24 (35.29)		
Income, RMB/year				0.577	0.749
< 15,000	230 (18.30)	216 (18.17)	14 (20.59)		
15,000~	288 (22.91)	271 (22.79)	17 (25.00)		
> 30,000	739 (58.79)	702 (59.04)	37 (54.41)		
BMI, kg/m^2^	24.16 ± 4.04	24.10 ± 4.05	25.20 ± 3.73	2.345	0.147
Hypertension, %				3.498	0.061
Yes	298 (23.71)	275 (23.13)	23 (33.82)		
No	959 (76.29)	914 (76.87)	45 (66.18)		
Family history of diabetes, %				2.421	0.120
Yes	542 (43.12)	506 (42.56)	36 (52.94)		
No	715 (56.88)	683 (57.44)	32 (47.06)		
Tea consumption, %				< 0.001	0.999
Never	984 (78.28)	931 (78.30)	53 (77.94)		
Ever or current	273 (21.72)	258 (21.70)	15 (22.06)		
Smoking status, %				2.241	0.134
Never	1004 (79.87)	955 (80.32)	49 (72.06)		
Ever or current	253 (20.13)	234 (19.68)	19 (27.94)		
Alcohol consumption, %				6.096	0.014
Never	912 (72.55)	872 (73.34)	40 (58.82)		
Ever or current	345 (27.45)	317 (26.66)	28 (41.18)		
Dietary patterns				2.094	0.351
Balanced	58 (4.61)	55 (4.63)	3 (4.41)		
General	690 (54.89)	647 (54.42)	43 (63.24)		
Unbalanced	509 (40.50)	487 (40.95)	22 (32.35)		
Physical activity, %				0.999	0.607
Low	610 (48.53)	573 (48.19)	37 (54.41)		
Moderate	545 (43.36)	519 (43.65)	26 (38.24)		
High	102 (8.11)	97 (8.16)	5 (7.35)		
Abnormal TG, %	465 (36.99)	437 (36.75)	28 (41.17)	0.367	0.545
Abnormal TC, %	576 (45.82)	534 (44.91)	42 (61.76)	6.696	0.010
Abnormal HDL-C, %	488 (38.82)	459 (38.60)	29 (42.65)	0.289	0.591
Abnormal LDL-C, %	487 (38.74)	453 (38.10)	34 (50.00)	3.353	0.067
Dyslipidemia, %	967 (76.93)	908 (76.37)	59 (86.76)	3.354	0.067
Abnormal CVAI, %	404 (32.14)	364 (30.61)	40 (58.82)	22.193	< 0.001

Values were expressed as mean (SD) or n (%). The differences between groups were compared by chi-square tests or t-tests (*P* value < 0.05 indicates significance). BMI: Body mass index; CVAI: Chinese visceral adiposity index; HDL-C: High-density lipoprotein cholesterol; LDL-C: Low-density lipoprotein cholesterol; T2DM: Type 2 diabetes mellitus; TG: Triglyceride; TC: Total cholesterol

### Associations between metabolic indicators and incident T2DM

During a median follow-up of 3.92 years, corresponding to 4,795.55 person-years (PYs), there was an incidence rate of 14.18 per 1,000 PYs. Table 2 presents the associations between lipid-related metabolic indicators and the risk of incident T2DM. In the fully adjusted model (Model 3), several indicators were significantly associated with an increased risk of T2DM. Specifically, TyG (HR = 1.758, 95% CI: 1.182–2.616), TyG-BMI (HR = 1.007, 95% CI: 1.002–1.013), TyG-WHtR (HR = 2.108, 95% CI: 1.468–3.027), TyG-WC (HR = 1.005, 95% CI: 1.003–1.007), CVAI (HR = 1.013, 95% CI: 1.006–1.020), and HOMA-IR (HR = 1.195, 95% CI: 1.038–1.376) were all positively associated with T2DM risk. In contrast, traditional lipid measures such as TG and TC were attenuated after full adjustment and were no longer statistically significant in Model 3. HDL-C and LDL-C showed no significant associations with T2DM risk across all models.

**Table 2:** Hazard ratios (HRs) and 95% confidence intervals (95% CIs) for incident T2DM in relation to lipid profiles

Metabolic factors	HR (95%CI)		
	Model 1	Model 2	Model 3
TC	1.296 (1.038, 1.618) *	1.267 (1.021, 1.573) *	1.242 (0.992, 1.556)
TG	1.134 (1.003, 1.282) *	1.105 (0.979, 1.247)	1.106 (0.973, 1.259)
TyG	1.900 (1.293, 2.792) *	1.763 (1.201, 2.586) *	1.758 (1.182, 2.616) *
TyG-BMI	1.007 (1.003, 1.012) *	1.007 (1.002, 1.012) *	1.007 (1.002, 1.013) *
TyG-WHtR	2.274 (1.637, 3.157) **	2.064 (1.466, 2.905) **	2.108 (1.468, 3.027) **
TyG-WC	1.005 (1.003, 1.007) **	1.005 (1.003, 1.007) **	1.005 (1.003, 1.007) **
CVAI	1.014 (1.008, 1.020) **	1.012 (1.005, 1.018) **	1.013 (1.006, 1.020) **
HDL-C	0.807 (0.368, 1.768)	0.834 (0.373, 1.865)	0.819 (0.360, 1.864)
LDL-C	1.211 (0.926, 1.583)	1.204 (0.923, 1.571)	1.176 (0.896, 1.544)
HOMA-IR	1.156 (1.001, 1.336) *	1.198 (1.043, 1.376) *	1.195 (1.038, 1.376) *

TC: Total cholesterol; TG: Triglyceride; TyG: Triglyceride-glucose; TyG-BMI: Triglyceride-glucose body mass index; TyG-WHtR: Triglyceride-glucose waist-to-height ratio; TyG-WC: Triglyceride-glucose waist circumference; CVAI: Chinese visceral adiposity index; HDL-C: High-density lipoprotein cholesterol; LDL-C: Low-density lipoprotein cholesterol; HOMA-IR: Homeostasis model assessment of insulin resistance

*P < 0.05, **P < 0.001

Model 1: Not adjusted

Model 2: Adjusted for age and gender

Model 3: Adjusted for age, gender, education, occupation, income, hypertension, family history of diabetes, smoking, alcohol consumption, tea consumption, physical activity, dietary patterns

The within-family analysis results are shown in Table 3. After controlling for shared familial factors, several associations remained statistically significant in the fully adjusted model. TyG (HR = 1.990, 95% CI: 1.052–3.767), TyG-WHtR (HR = 1.812, 95% CI: 1.049–3.132), TyG-WC (HR = 1.005, 95% CI: 1.001–1.009), CVAI (HR = 1.011, 95% CI: 1.001–1.022), and HOMA-IR (HR = 1.412, 95% CI: 1.005–1.982) remained significantly associated with increased T2DM risk. However, the associations for traditional lipid indicators such as TG and TC were attenuated and no longer statistically significant after accounting for familial clustering.

**Table 3:** Within-family analysis of Hazard ratios (HRs) and 95% confidence intervals (95% CIs) for incident T2DM in relation to lipid profiles

Metabolic factors	HR (95%CI)		
	Model 1	Model 2	Model 3
TC	1.270 (0.939, 1.717)	1.268 (0.928, 1.733)	1.215 (0.851, 1.736)
TG	1.325 (1.035, 1.696) *	1.311 (1.015, 1.694) *	1.266 (0.947, 1.692)
TyG	2.209 (1.264, 3.862) *	2.167 (1.228, 3.823) *	1.990 (1.052, 3.767) *
TyG-BMI	1.007 (0.999, 1.015)	1.007 (0.999, 1.015)	1.008 (0.999, 1.018)
TyG-WHtR	1.812 (1.165, 2.817) *	1.696 (1.085, 2.651) *	1.812 (1.049, 3.132) *
TyG-WC	1.004 (1.001, 1.007) *	1.004 (1.001, 1.007) *	1.005 (1.001, 1.009) *
CVAI	1.011 (1.003, 1.018) *	1.009 (1.001, 1.017) *	1.011 (1.001, 1.022) *
HDL-C	0.724 (0.272, 1.929)	0.709 (0.264, 1.905)	0.789 (0.254, 2.450)
LDL-C	1.147 (0.831, 1.583)	1.148 (0.824, 1.601)	1.063 (0.730, 1.548)
HOMA-IR	1.232 (0.939, 1.616)	1.286 (0.973, 1.701)	1.412 (1.005, 1.982) *

Abbreviations are as defined in Table 2

*P < 0.05, **P < 0.001

Model 1: Not adjusted

Model 2: Adjusted for age and gender

Model 3: Adjusted for age, gender, education, occupation, income, hypertension, family history of diabetes, smoking, alcohol consumption, tea consumption, physical activity, dietary patterns

### Quartile-based and trend analysis

As shown in Table 4, quartile-based analyses further demonstrated dose-response relationships between key metabolic indicators and incident T2DM risk. For TyG, participants in higher quartiles had significantly increased risks compared with the lowest quartile, and a significant trend was observed across quartiles (*P*_trend_ = 0.023 in Model 3). Similarly, TyG-WHtR and TyG-WC showed progressively increased risks across quartiles, with significant trends observed in the fully adjusted model (*P*_trend_ = 0.003 and *P*_trend_ < 0.001). CVAI exhibited the strongest gradient, with participants in the highest quartile having a markedly increased risk of T2DM (HR = 6.455, 95% CI: 2.228–18.708), and a highly significant dose-response relationship (*P*_trend_ < 0.001). For HOMA-IR, although the associations across quartiles were not statistically significant, a borderline trend was observed (*P*_trend_ = 0.059).

**Table 4:** Quartile-based and trend analysis of key metabolic indicators with incident T2DM

Metabolic factors	Model	HR (95%CI)				*P* trend
		Q1	Q2	Q3	Q4	
TyG	Model1	1.000	3.408 (1.440, 8.065) *	2.493 (1.013, 6.132) *	3.752 (1.586, 8.877) *	0.010
	Model 2	1.000	2.985 (1.258, 7.084) *	2.413 (0.981, 5.932)	3.278 (1.384, 7.768) *	0.021
	Model 3	1.000	3.077 (1.285, 7.370) *	2.540 (1.017, 6.340) *	3.325 (1.381, 8.007) *	0.023
TyG-WHtR	Model1	1.000	2.302 (0.868, 6.107)	4.096 (1.651, 10.163) **	4.844 (1.969, 11.916) **	< 0.001
	Model 2	1.000	2.067 (0.777, 5.499)	3.366 (1.342, 8.441) *	3.844 (1.540, 9.599) *	0.002
	Model 3	1.000	2.146 (0.792, 5.812)	3.472 (1.350, 8.929) *	3.940 (1.520, 10.211) *	0.003
TyG-WC	Model1	1.000	1.439 (0.543, 3.813)	3.547 (1.506, 8.353) *	4.433 (1.914, 10.268) **	< 0.001
	Model 2	1.000	1.273 (0.478, 3.391)	2.929 (1.227, 6.989) *	3.653 (1.547, 8.629) *	< 0.001
	Model 3	1.000	1.418 (0.522, 3.854)	3.041 (1.245, 7.428) *	3.725 (1.518, 9.139) *	< 0.001
CVAI	Model1	1.000	2.363 (0.827, 6.749)	3.708 (1.368, 10.049) *	6.943 (2.690, 17.916) **	< 0.001
	Model 2	1.000	2.166 (0.754, 6.226)	3.077 (1.111, 8.522) *	5.196 (1.915, 14.097) *	< 0.001
	Model 3	1.000	2.509 (0.851, 7.396)	3.829 (1.317, 11.132) *	6.455 (2.228, 18.708) **	< 0.001
HOMA-IR	Model1	1.000	0.758 (0.354, 1.622)	1.162 (0.588, 2.297)	1.396 (0.714, 2.730)	0.174
	Model 2	1.000	0.818 (0.382, 1.751)	1.220 (0.617, 2.416)	1.726 (0.875, 3.405)	0.053
	Model 3	1.000	0.803 (0.370, 1.740)	1.233 (0.612, 2.485)	1.717 (0.848, 3.479)	0.059

TyG: Triglyceride-glucose; TyG-WHtR: Triglyceride-glucose waist-to-height ratio; TyG-WC: Triglyceride-glucose waist circumference; CVAI: Chinese visceral adiposity index; HOMA-IR: Homeostasis model assessment of insulin resistance

*P < 0.05, **P < 0.001

Model 1: Not adjusted

Model 2: Adjusted for age and gender

Model 3: Adjusted for age, gender, education, occupation, income, hypertension, family history of diabetes, smoking, alcohol consumption, tea consumption, physical activity, dietary patterns

## Discussion

This diabetes family cohort study comprehensively examined the associations of multiple lipid-related metabolic indicators with incident T2DM and further evaluated the robustness of these associations through within-family and trend analyses. Overall, the present study revealed that several composite metabolic indicators, including TyG, TyG-BMI, TyG-WHtR, TyG-WC, CVAI, and HOMA-IR, were significantly associated with an increased risk of T2DM. The within-family analysis further showed that the associations for TyG, TyG-WHtR, TyG-WC, CVAI, and HOMA-IR remained significant after accounting for shared familial factors, suggesting that these indicators may better capture individual-level metabolic risk beyond familial aggregation. In addition, quartile-based and trend analyses demonstrated clear dose-response relationships for TyG, TyG-WHtR, TyG-WC, and CVAI. These findings provide useful evidence that composite metabolic indicators were consistently associated with incident T2DM, whereas the associations for conventional lipid parameters were attenuated after multivariable adjustment.

An increasing number of studies have suggested that composite metabolic indicators may outperform conventional single lipid measures in identifying diabetes risk. A study in middle-aged and elderly Chinese adults showed that TyG-related indices and CVAI had better predictive performance for T2DM than conventional anthropometric indicators [32]. Consistently, cohort studies further reported that TyG-WHtR, TyG-BMI, and other obesity- or lipid-related composite indices were significantly associated with incident diabetes and showed relatively consistent associations across different populations [34–36]. Additional evidence from Chinese community-based and hypertensive populations also supported the superiority of integrated metabolic indicators over isolated anthropometric or lipid parameters in diabetes risk stratification [14, 36, 37]. These previous findings were largely in line with our results, in which TyG, TyG-related indices, CVAI, and HOMA-IR showed more stable and significant associations with incident T2DM than conventional lipid measures. By contrast, we did not observe independent associations for traditional lipid measures such as TG, TC, HDL-C, or LDL-C after full adjustment. Evidence for these single markers remains less consistent. Several studies have reported positive associations between dyslipidemia, LDL-C, non-HDL-C, or low HDL-C and incident T2DM [20, 38], whereas the magnitude and stability of these associations often varied by study population, baseline metabolic status, and adjustment strategy [39, 40]. In a rural Chinese cohort, higher TG and TC levels, as well as TC/HDL-C and TG/HDL-C ratios, were statistically associated with increased T2DM risk [41]. Similarly, a prospective cohort study in northwest China found significant associations for abnormal TC, TG, and HDL-C, whereas LDL-C was not independently associated after multivariable adjustment [42]. Another rural Chinese cohort showed that cumulative TG and HDL burdens were associated with incident T2DM [43], suggesting that a single baseline lipid measurement may not fully capture long-term lipid-related metabolic disturbances. Notably, participants in our study were recruited from diabetes families and shared a relatively high familial susceptibility to T2DM, which may have reduced the ability of individual lipid parameters to distinguish subsequent risk. One plausible explanation is that single lipid fractions capture only one aspect of metabolic disturbance, whereas composite indicators simultaneously incorporate information on dyslipidemia, glucose metabolism, and central adiposity, thereby better reflecting the complex pathophysiological processes underlying T2DM [32, 34, 35]. In addition, blood lipid traits are substantially influenced by inherited factors. Recent genetic studies and reviews have highlighted the strong heritability and polygenic architecture of lipid phenotypes overall, particularly for LDL-C and HDL-C [44–46]. Therefore, in our family-based study, no significant associations were observed for HDL-C or LDL-C, possibly because these two markers are more strongly influenced by shared genetic background, which may attenuate the independent predictive value of a single baseline measurement for future T2DM. The relatively small sample size was not enough to find the real associations for single indicators in this study, which may be another plausible explanation.

The additional analyses further strengthened the interpretation of the main findings. In the within-family analysis, the associations for TyG, TyG-WHtR, TyG-WC, CVAI, and HOMA-IR remained statistically significant, suggesting that these indicators captured individual-level metabolic risk beyond shared familial background. This pattern was consistent with previous studies showing that composite or insulin resistance-related markers generally provide more robust information on diabetes risk than single lipid parameters alone [47–49]. In contrast, the attenuation of TG, TC, and TyG-BMI in the within-family models implied that the associations of these markers may be more susceptible to shared genetic or household-level influences, such as common dietary habits, adiposity patterns, and inherited metabolic susceptibility. Taken together, these findings suggested that composite metabolic indicators may better reflect diabetes-related metabolic dysregulation that was less confounded by familial aggregation. The quartile-based and trend analyses provided further evidence of dose-response relationships. TyG, TyG-WHtR, TyG-WC, and CVAI showed progressively higher risks of incident T2DM across increasing quartiles, with CVAI exhibiting the strongest gradient, which was biologically plausible and also agreed with previous reports indicating that TyG-related indices, CVAI, and other integrated metabolic markers may more comprehensively capture the joint effects of abdominal adiposity, dyslipidemia, and insulin resistance than conventional lipid measures alone [50–52]. By comparison, the trend for HOMA-IR was only borderline significant in this study. Given that HOMA-IR is a well-established marker of insulin resistance and that recent reviews continue to support the predictive value of insulin resistance–related biomarkers for metabolic outcomes [33, 53, 54], the weaker significance in our quartile analysis may be related to reduced statistical power after categorization, especially in the context of the relatively small number of incident cases in our cohort. Overall, these additional analyses supported the robustness and biological plausibility of our primary findings.

Previous family-based metabolic cohort studies have shown that insulin resistance-related traits, adiposity, and lipid abnormalities are associated with subsequent T2DM development among individuals with familial susceptibility [55–57]. Building on these findings, our study evaluated multiple lipid-related metabolic indicators in a diabetes family cohort and found that TyG-related indices, CVAI, and HOMA-IR remained associated with incident T2DM after accounting for familial clustering. These findings further support the potential relevance of composite metabolic markers in assessing T2DM risk in high-risk family populations.

This study has several strengths. First, by simultaneously evaluating conventional lipid markers, TyG-related indices, CVAI, and HOMA-IR within the same family cohort, we were able to directly compare the relative value of traditional and composite metabolic indicators. Second, the use of both within-family analysis and quartile-based trend analysis enhanced the robustness of the findings and provided additional support beyond the primary Cox models. Several limitations should also be acknowledged. First, the overall sample size was relatively modest, and only 68 incident T2DM cases occurred during follow-up. This limited number of events may have reduced statistical power, particularly in the within-family and quartile-based analyses, and may partly explain the wide confidence intervals and borderline findings for some indicators such as HOMA-IR. Also, categorizing continuous indicators into quartiles may have further reduced statistical efficiency with unstable estimates, which should be interpreted cautiously. No formal correction for multiple comparisons was performed in this study, so the possibility of chance findings due to multiple testing could not be completely excluded. Second, the metabolic indicators used in the analyses were measured once at baseline, which may introduce measurement bias, and we were therefore unable to account for longitudinal changes in lipid metabolism or insulin resistance over time. Furthermore, follow-up HbA1c measurements were available for only a small proportion of participants (16.87%), which may have resulted in missed cases with elevated HbA1c but normal FPG levels and consequently underestimated the incidence of T2DM. Moreover, regression dilution bias due to biological variability and measurement error could not be completely excluded. Third, although multiple covariates were adjusted for and familial clustering was considered in the analyses, residual confounding from unmeasured genetic, environmental, or lifestyle-related factors could not be ruled out. In addition, subjects excluded were different to the followed in many baseline characteristics including age and occupation, which may make a select bias in this study. Finally, because this study was conducted in a Chinese family-based cohort, caution is warranted when generalizing the findings to other populations. These findings called to be validated in larger multicenter family-based cohorts in future, where the associations across different baseline glucose metabolic states should be examined, and repeated measurements of metabolic indicators incorporated to better characterize dynamic metabolic changes.

## Conclusion

In conclusion, this diabetes family-based cohort study confirmed the associations of several composite metabolic indicators, including TyG-related indices, CVAI, and HOMA-IR, with incident T2DM. Moving forward, greater attention should be paid to the assessment and management of metabolic dysfunction, particularly using composite indicators that integrate lipid metabolism, adiposity, and insulin resistance. In addition, composite indicators should be prioritized for the early identification of individuals at high risk of T2DM, so as to support the implementation of targeted prevention and intervention strategies.

## Supplementary Information

Below is the link to the electronic supplementary material.

Supplementary Material 1

## Data Availability

The materials might be made available upon request, some restrictions will apply.

## Abbreviations

BMI: Body mass index
CVAI: Chinese visceral adiposity index
DBP: Diastolic blood pressure
FINS: Fasting insulin
FPG: Fasting plasma glucose
HbA1c: Hemoglobin A1c
HDL-C: High-density lipoprotein cholesterol
HC: Hip circumference
HOMA-IR: Homeostasis model assessment of insulin resistance
IPAQ: International Physical Activity Questionnaire
LDL-C: Low-density lipoprotein cholesterol
SBP: Systolic blood pressure
T2DM: Type 2 diabetes mellitus
TC: Total cholesterol
TG: Triglyceride
TyG: Triglyceride-glucose index
TyG-BMI: Triglyceride-glucose body mass index
TyG-WC: Triglyceride-glucose waist circumference
TyG-WHtR: Triglyceride-glucose waist-to-height ratio
WC: Waist circumference

## References

[1] Genitsaridi I, Salpea P, Salim A, et al. 11th edition of the IDF Diabetes Atlas: global, regional, and national diabetes prevalence estimates for 2024 and projections for 2050 [J]. Lancet Diabetes Endocrinol. 2026;14(2):149–56. 10.1016/S2213-8587(25)00299-2 41412135

[2] Collaborators GBDD. Global, regional, and national burden of diabetes from 1990 to 2021, with projections of prevalence to 2050: a systematic analysis for the Global Burden of Disease Study 2021 [J]. Lancet. 2023;402(10397):203–34. 10.1016/S0140-6736(23)01301-6 PMC10364581 37356446

[3] Xu Y, Lu J, Li M, et al. Diabetes in China part 1: epidemiology and risk factors [J]. Lancet Public Health. 2024;9(12):e1089–97. 10.1016/S2468-2667(24)00250-0 39579774

[4] Zhou YC, Liu JM, Zhao ZP, et al. The national and provincial prevalence and non-fatal burdens of diabetes in China from 2005 to 2023 with projections of prevalence to 2050 [J]. Mil Med Res. 2025;12(1):28. 10.1186/s40779-025-00615-1 PMC12128495 40457495

[5] Jahdkaran M, Sistanizad M. From lipids to glucose: Investigating the role of dyslipidemia in the risk of insulin resistance [J]. J Steroid Biochem Mol Biol. 2025;250:106744. 10.1016/j.jsbmb.2025.106744 40158704

[6] Li X, Sun M, Yang Y, et al. Predictive Effect of Triglyceride Glucose-Related Parameters, Obesity Indices, and Lipid Ratios for Diabetes in a Chinese Population: A Prospective Cohort Study [J]. Front Endocrinol (Lausanne). 2022;13:862919. 10.3389/fendo.2022.862919 PMC9007200 35432185

[7] Zhu Y, He Y, Fang S, et al. Predictive performance of cardiometabolic indices for prediabetes progression to diabetes and reversion to normoglycemia in adults: a cohort study [J]. Front Nutr. 2026;13:1784247. 10.3389/fnut.2026.1784247 PMC13215993 42221790

[8] D’elia L, Rendina D, Iacone R, et al. Triglyceride-glucose index and new-onset type 2 diabetes mellitus in middle-aged men [J]. Metabolites. 2025;15(8):537. 10.3390/metabo15080537 PMC12388705 40863155

[9] Wang Y, Cheng T, Zhang T, et al. Association of triglyceride glucose index with incident diabetes among individuals with normal fasting triglycerides and fasting plasma glucose values: a general population-based retrospective cohort study [J]. Front Endocrinol (Lausanne). 2025;16:1598171. 10.3389/fendo.2025.1598171 PMC12206639 40589514

[10] Chen H, Chen Q, Xia L, et al. Threshold effect and age interaction of TyG index on diabetes incidence in normolipidemic population: a multicenter cohort study [J]. Front Endocrinol (Lausanne). 2025;16:1645344. 10.3389/fendo.2025.1645344 PMC12620258 41255531

[11] Qiao Q, Liang K, Wang C, et al. J-shaped association of the triglyceride glucose-body mass index with new-onset diabetes [J]. Sci Rep. 2024;14(1):13882. 10.1038/s41598-024-64784-0 PMC11180648 38880800

[12] Xuan W, Liu D, Zhong J, et al. Impacts of Triglyceride Glucose-Waist to Height Ratio on Diabetes Incidence: A Secondary Analysis of A Population-Based Longitudinal Data [J]. Front Endocrinol (Lausanne). 2022;13:949831. 10.3389/fendo.2022.949831 PMC9354460 35937805

[13] Wen S, Huang X, Huang Z, et al. The association of changes in the Chinese visceral adiposity index and cardiometabolic diseases: a cohort study [J]. Diabetol Metab Syndr. 2024;16(1):228. 10.1186/s13098-024-01460-3 PMC11401298 39272152

[14] Jin X, Wei Y, Mo Y, et al. Associations of obesity and novel lipid indicators in the risk of type 2 diabetes mellitus in Chinese elderly hypertensive patients [J]. Front Endocrinol (Lausanne). 2025;16:1475323. 10.3389/fendo.2025.1475323 PMC11996637 40235662

[15] Stefan AG, Clenciu D, Vladu IM, et al. Relationship between insulin resistance indicators and type 2 diabetes mellitus in Romania [J]. Int J Mol Sci. 2025;26(20):9888. 10.3390/ijms26209888 PMC12563221 41155183

[16] Lu M, Guo J, Yang P, et al. Insulin resistance markers HOMA-IR, TyG and TyG-BMI index in relation to heart failure risk: NHANES 2011–2016 [J]. PLoS ONE. 2025;20(9):e0331740. 10.1371/journal.pone.0331740 PMC12478908 41021613

[17] Meigs JB, Cupples LA, WILSON PW. Parental transmission of type 2 diabetes: the Framingham Offspring Study [J]. Diabetes. 2000;49(12):2201–7. 10.2337/diabetes.49.12.2201 11118026

[18] Zaccardi F, O’donovan G, Webb DR, et al. Cardiorespiratory fitness and risk of type 2 diabetes mellitus: A 23-year cohort study and a meta-analysis of prospective studies [J]. Atherosclerosis. 2015;243(1):131–7. 10.1016/j.atherosclerosis.2015.09.016 26386209

[19] Szili-Torok T, Bakker SJL, Tietge UJF. Normal fasting triglyceride levels and incident type 2 diabetes in the general population [J]. Cardiovasc Diabetol. 2022;21(1):111. 10.1186/s12933-022-01530-8 PMC9206357 35717188

[20] Huang J, Lin H, Wang S, et al. Association between serum LDL-C concentrations and risk of diabetes: A prospective cohort study [J]. J Diabetes. 2023;15(10):881–9. 10.1111/1753-0407.13440 PMC10590678 37461165

[21] Seo IH, Son DH, Lee HS, et al. Non-HDL cholesterol as a predictor for incident type 2 diabetes in community-dwelling adults: longitudinal findings over 12 years [J]. Transl Res. 2022;243:52–9. 10.1016/j.trsl.2021.12.008 34979322

[22] American Diabetes Association Professional Practice C. 2. Classification and Diagnosis of Diabetes: Standards of Medical Care in Diabetes-2022 [J]. Diabetes Care. 2022;45(Suppl 1):S17–38. 10.2337/dc22-S002 34964875

[23] Craig CL, Marshall AL, Sjostrom M, et al. International physical activity questionnaire: 12-country reliability and validity [J]. Med Sci Sports Exerc. 2003;35(8):1381–95. 10.1249/01.MSS.0000078924.61453.FB 12900694

[24] Reguant-Closa A, Pedolin D, Herrmann M, et al. Review of Diet Quality Indices that can be Applied to the Environmental Assessment of Foods and Diets [J]. Curr Nutr Rep. 2024;13(2):351–62. 10.1007/s13668-024-00540-0 PMC11133024 38625631

[25] Mcevoy JW, Mccarthy CP, Bruno RM, et al. 2024 ESC Guidelines for the management of elevated blood pressure and hypertension [J]. Eur Heart J. 2024;45(38):3912–4018. 10.1093/eurheartj/ehae178 39210715

[26] Li JJ, Zhao SP, Zhao D, et al. 2023 Chinese guideline for lipid management [J]. Front Pharmacol. 2023;14:1190934. 10.3389/fphar.2023.1190934 PMC10498001 37711173

[27] Simental-Mendia LE, Rodriguez-Moran M, Guerrero-Romero F. The product of fasting glucose and triglycerides as surrogate for identifying insulin resistance in apparently healthy subjects [J]. Metab Syndr Relat Disord. 2008;6(4):299–304. 10.1089/met.2008.0034 19067533

[28] Park B, Lee HS, Lee YJ. Triglyceride glucose (TyG) index as a predictor of incident type 2 diabetes among nonobese adults: a 12-year longitudinal study of the Korean Genome and Epidemiology Study cohort [J]. Transl Res. 2021;228:42–51. 10.1016/j.trsl.2020.08.003 32827706

[29] Er LK, Wu S, Chou HH, et al. Triglyceride Glucose-Body Mass Index Is a Simple and Clinically Useful Surrogate Marker for Insulin Resistance in Nondiabetic Individuals [J]. PLoS ONE. 2016;11(3):e0149731. 10.1371/journal.pone.0149731 PMC4773118 26930652

[30] Zheng D, Cai J, Xu S, et al. The association of triglyceride-glucose index and combined obesity indicators with chest pain and risk of cardiovascular disease in American population with pre-diabetes or diabetes [J]. Front Endocrinol (Lausanne). 2024;15:1471535. 10.3389/fendo.2024.1471535 PMC11412814 39309107

[31] Xia MF, Chen Y, Lin HD, et al. A indicator of visceral adipose dysfunction to evaluate metabolic health in adult Chinese [J]. Sci Rep. 2016;6:38214. 10.1038/srep38214 PMC5131270 27905531

[32] Zhang X, Wang Y, Li Y, et al. Optimal obesity- and lipid-related indices for predicting type 2 diabetes in middle-aged and elderly Chinese [J]. Sci Rep. 2024;14(1):10901. 10.1038/s41598-024-61592-4 PMC11091178 38740846

[33] Sasaki N, Ueno Y, Higashi Y. Indicators of insulin resistance in clinical practice [J]. Hypertens Res. 2024;47(4):978–80. 10.1038/s41440-023-01566-7 38177290

[34] Dou JH, Guo FS, Guo C, et al. Triglyceride glucose-waist height ratio index is a promising and robust glucose and lipid metabolism indicator for predicting long-term type 2 diabetes: insights from a Japanese prospective cohort data from 12 years [J]. Diabetes Res Clin Pract. 2025;227:112398. 10.1016/j.diabres.2025.112398 40749948

[35] Shen X, Nan J, Mou L, et al. Combination of triglyceride-glucose index and waist-to-height ratio as a predictor of all-cause and cardiovascular mortality in adults with diabetes or prediabetes: a nationwide prospective cohort study [J]. Cardiovasc Diagn Ther. 2025;15(5):937–54. 10.21037/cdt-2025-206 PMC12596460 41216255

[36] Wang Y, Zhang X, Li Y, et al. Obesity- and lipid-related indices as a predictor of type 2 diabetes in a national cohort study [J]. Front Endocrinol (Lausanne). 2023;14:1331739. 10.3389/fendo.2023.1331739 PMC10864443 38356678

[37] Huang Z, Zhang X, Sun D, et al. Triglyceride to high-density lipoprotein cholesterol ratio is associated with diabetes incidence in non-obese individuals with normoglycemia: a retrospective cohort study based on individuals from East Asia [J]. Front Endocrinol (Lausanne). 2024;15:1442731. 10.3389/fendo.2024.1442731 PMC11586199 39588337

[38] Peng J, Zhao F, Yang X, et al. Association between dyslipidemia and risk of type 2 diabetes mellitus in middle-aged and older Chinese adults: a secondary analysis of a nationwide cohort [J]. BMJ Open. 2021;11(5):e042821. 10.1136/bmjopen-2020-042821 PMC8154929 34035089

[39] Han M, Shen Y, Guo X, et al. Association between non-high-density lipoprotein cholesterol and type 2 diabetes: a systematic review and meta-analysis of cohort studies [J]. Endocr J. 2025;72(1):43–51. 10.1507/endocrj.EJ24-0189 PMC11778345 39313371

[40] Yan Z, Xu Y, Li K, et al. Association between high-density lipoprotein cholesterol and type 2 diabetes mellitus: dual evidence from NHANES database and Mendelian randomization analysis [J]. Front Endocrinol (Lausanne). 2024;15:1272314. 10.3389/fendo.2024.1272314 PMC10917910 38455653

[41] Zhang M, Zhou J, Liu Y, et al. Risk of type 2 diabetes mellitus associated with plasma lipid levels: The rural Chinese cohort study [J]. Diabetes Res Clin Pract. 2018;135:150–7. 10.1016/j.diabres.2017.11.011 29155120

[42] Wang MZ, Dai T, Zheng S, et al. The Combined Effect of Dyslipidemia on the Incidence of Type 2 Diabetes: A Prospective Cohort Study in Northwest of China [J]. Biomed Environ Sci. 2021;34(10):814–8. 10.3967/bes2021.110 34782047

[43] Wang Q, Xie T, Zhang T, et al. The Role of Changes in Cumulative Lipid Parameter Burden in the Pathogenesis of Type 2 Diabetes Mellitus: A Cohort Study of People Aged 35–65 Years in Rural China [J]. Diabetes Metab Syndr Obes. 2022;15:1831–43. 10.2147/DMSO.S363692 PMC9208634 35733642

[44] Dron JS, Natarajan P, Peloso GM. The breadth and impact of the Global Lipids Genetics Consortium [J]. Curr Opin Lipidol. 2025;36(2):61–70. 10.1097/MOL.0000000000000966 PMC11888832 39602359

[45] Kalwick M, Roth M. A comprehensive review of the genetics of dyslipidemias and risk of atherosclerotic cardiovascular disease [J]. Nutrients. 2025;17(4):659. 10.3390/nu17040659 PMC11858766 40004987

[46] Chermon D, Birk R, Polygenic, risk. modifiable lifestyle behaviors, and metabolic factors: associations with HDL-C, triglyceride levels, and cardiovascular risk [J]. Nutrients. 2025;17(13):2244. 10.3390/nu17132244 PMC12252312 40647348

[47] Navarro-Gonzalez D, Sanchez-Inigo L, Pastrana-Delgado J, et al. Triglyceride-glucose index (TyG index) in comparison with fasting plasma glucose improved diabetes prediction in patients with normal fasting glucose: The Vascular-Metabolic CUN cohort [J]. Prev Med. 2016;86:99–105. 10.1016/j.ypmed.2016.01.022 26854766

[48] Xia MF, Lin HD, Chen LY, et al. Association of visceral adiposity and its longitudinal increase with the risk of diabetes in Chinese adults: A prospective cohort study [J]. Diabetes Metab Res Rev. 2018;34(7):e3048. 10.1002/dmrr.3048 30035847

[49] Ramdas Nayak VK, Nayak KR, Vidyasagar S, et al. Predictive performance of traditional and novel lipid combined anthropometric indices to identify prediabetes [J]. Diabetes Metab Syndr. 2020;14(5):1265–72. 10.1016/j.dsx.2020.06.045 32688243

[50] Shangguan Q, Liu Q, Yang R, et al. Predictive value of insulin resistance surrogates for the development of diabetes in individuals with baseline normoglycemia: findings from two independent cohort studies in China and Japan [J]. Diabetol Metab Syndr. 2024;16(1):68. 10.1186/s13098-024-01307-x PMC10943817 38491516

[51] Jiang K, Luan H, Pu X, et al. Association Between Visceral Adiposity Index and Insulin Resistance: A Cross-Sectional Study Based on US Adults [J]. Front Endocrinol (Lausanne). 2022;13:921067. 10.3389/fendo.2022.921067 PMC9353944 35937809

[52] Wei J, Liu X, Xue H, et al. Comparisons of visceral adiposity index, body shape index, body mass index and waist circumference and their associations with diabetes mellitus in adults [J]. Nutrients;2019:11(7):1580. 10.3390/nu11071580 PMC6683101 31336951

[53] Gonzalez-Gonzalez JG, Violante-Cumpa JR, Zambrano-Lucio M, et al. Homa-IR as a predictor of health outcomes in patients with metabolic risk factors: a systematic review and meta-analysis [J]. High Blood Press Cardiovasc Prev; 2022;29:547–64. 6. 10.1007/s40292-022-00542-5 36181637

[54] Lee J, Kim MH, Jang JY, et al. Assessment HOMA as a predictor for new onset diabetes mellitus and diabetic complications in non-diabetic adults: a KoGES prospective cohort study [J]. Clin Diabetes Endocrinol. 2023;9(1):7. 10.1186/s40842-023-00156-3 PMC10652621 37974292

[55] Hanley AJ, Wagenknecht LE, Norris JM, et al. Insulin resistance, beta cell dysfunction and visceral adiposity as predictors of incident diabetes: the Insulin Resistance Atherosclerosis Study (IRAS) Family study [J]. Diabetologia. 2009;52(10):2079–86. 10.1007/s00125-009-1464-y PMC3992852 19641896

[56] Wu J, Wang X, Chen H, et al. Type 2 diabetes risk and lipid metabolism related to the pleiotropic effects of an ABCB1 variant: a Chinese family-based cohort study [J]. Metabolites. 2022;12(9):875. 10.3390/metabo12090875 PMC9502507 36144279

[57] Sadeghi E, Hosseini SM, Vossoughi M, et al. Association of Lipid Profile with Type 2 Diabetes in First-Degree Relatives: A 14-Year Follow-Up Study in Iran [J]. Diabetes Metab Syndr Obes. 2020;13:2743–50. 10.2147/DMSO.S259697 PMC7415448 32801820

