# Supplementary material for: Risk of type 2 diabetes mellitus associated with lipid-related metabolic indicators: a diabetes family cohort study in China
Source: BMC Endocr Disord. 2026 Aug 12;26:362. doi: 10.1186/s12902-026-02484-8 (PMC13746738; doi:10.1186/s12902-026-02484-8)
Supplement: Supplementary file 1 [file 12902_2026_2484_MOESM1_ESM.docx]

| **Supplementary Table 1.** Comparison of Baseline Characteristics Between Participants Included in and Excluded From the Final Analysis | | | | | |
| --- | --- | --- | --- | --- | --- |
| Characteristics | Total (*n*=1888) | No(*n*=1257) | Yes(*n*=631) | *χ*^2^/*t* | *P* *value* |
| Age, years | 49.39±18.47 | 52.19±16.02 | 43.80±21.53 |  | <0.001 |
| <60 | 1366 (72.35) | 881 (70.09) | 485 (76.86) | 9.303 | 0.002 |
| ≥60 | 522 (27.65) | 376 (29.91) | 146 (23.14) |  |  |
| Gender, % |  |  |  | 2.083 | 0.149 |
| Male | 757 (40.10) | 489 (38.90) | 268 (42.47) |  |  |
| Female | 1131 (59.90) | 768 (61.10) | 363 (57.53) |  |  |
| Education, years |  |  |  | 1.038 | 0.308 |
| <9 | 1295 (68.59) | 852 (67.78) | 443 (70.21) |  |  |
| ≥9 | 593 (31.41) | 405 (32.22) | 188 (29.79) |  |  |
| Marital status, % |  |  |  | 61.621 | <0.001 |
| Married | 1503 (79.61) | 1066 (84.81) | 437 (69.26) |  |  |
| Not married | 385 (20.39) | 191 (15.19) | 194 (30.74) |  |  |
| Occupation, % |  |  |  | 35.188 | <0.001 |
| Mainly intellectual work | 528 (27.97) | 297 (23.63) | 231 (36.61) |  |  |
| Mainly physical labor | 871 (46.13) | 613 (48.77) | 258 (48.89) |  |  |
| Unemployment or others | 489 (25.90) | 347 (27.60) | 142 (22.50) |  |  |
| Income, RMB/year |  |  |  | 1.437 | 0.487 |
| <15000 | 332 (17.58) | 230 (18.30) | 102 (16.16) |  |  |
| 15000~ | 432 (22.88) | 288 (22.91) | 144 (22.82) |  |  |
| >30000 | 1124 (59.54) | 739 (58.79) | 385 (61.01) |  |  |
| Hypertension, % |  |  |  | 7.959 | 0.005 |
| Yes | 411 (21.77) | 298 (23.71) | 113 (17.91) |  |  |
| No | 1477 (78.23) | 959 (76.29) | 518 (82.09) |  |  |
| Family history of diabetes, % |  |  |  | 3.998 | 0.046 |
| Yes | 783 (41.47) | 542 (43.12) | 241 (38.19) |  |  |
| No | 1105 (58.53) | 715 (56.88) | 390 (61.81) |  |  |
| Tea consumption, % |  |  |  | 1.125 | 0.289 |
| Never | 1492 (79.03) | 984 (78.28) | 508 (80.51) |  |  |
| Ever or current | 396 (20.97) | 273 (21.72) | 123 (19.49) |  |  |
| Smoking status, % |  |  |  | 3.728 | 0.053 |
| Never | 1532 (81.14) | 1004 (79.87) | 528 (83.68) |  |  |
| Ever or current | 356 (18.86) | 253 (20.13) | 103 (16.32) |  |  |
| Alcohol consumption, % |  |  |  | 5.815 | 0.016 |
| Never | 1403 (73.31) | 912 (72.55) | 491 (77.81) |  |  |
| Ever or current | 485 (25.69) | 345 (27.45) | 140 (22.19) |  |  |
| Dietary patterns |  |  |  | 0.448 | 0.351 |
| Balanced | 83 (4.40) | 58 (4.61) | 25 (3.96) |  |  |
| General | 1041 (55.14) | 690 (54.89) | 351 (55.63) |  |  |
| Unbalanced | 764 (40.46) | 509 (40.50) | 255 (40.41) |  |  |
| Physical activity, % |  |  |  | 0.808 | 0.668 |
| Low | 923 (48.89) | 610 (48.53) | 313 (49.60) |  |  |
| Moderate | 819 (43.38) | 545 (43.36) | 274 (43.42) |  |  |
| High | 146 (7.73) | 102 (8.11) | 44 (6.98) |  |  |

Values were expressed as mean (SD) or n (%). The differences between groups were compared by chi-square tests or t-tests (P value < 0.05 indicates significance).
